# Unlike PD-L1, PD-1 Is Downregulated on Partial Immune Cells in Type 2 Diabetes

**DOI:** 10.1155/2019/5035261

**Published:** 2019-03-17

**Authors:** Peng Sun, Qingyan Jin, Shengnan Nie, Shijie Jia, Yuanyuan Li, Xiaoxue Li, Fang Guo

**Affiliations:** ^1^Department of Intervention Oncology, Shandong Cancer Hospital and Institute, Shandong Academy of Medical Sciences, Jinan, Shandong, China; ^2^The Key Laboratory of Cardiovascular Remodeling and Function Research, Chinese Ministry of Education, Chinese National Health Commission and Chinese Academy of Medical Sciences, The State and Shandong Province Joint Key Laboratory of Translational Cardiovascular Medicine, Department of Cardiology, Qilu Hospital of Shandong University, Jinan, China; ^3^Operation Room of Intervention Oncology, Shandong Cancer Hospital and Institute, Shandong Academy of Medical Sciences, Jinan, Shandong, China

## Abstract

**Introduction:**

Type 2 diabetes is a worldwide disease which is associated with chronic, low-grade inflammation. The PD-1/PD-L1 pathway has been reported to be a negative regulatory element in immune homeostasis and to be involved in many diseases.

**Materials and Methods:**

Peripheral blood mononuclear cells (PBMCs) were obtained from type 2 diabetes patients (*n* = 23) and healthy donors (*n* = 20). The PD-L1 and PD-1 expressions on corresponding immune cells were evaluated by flow cytometry.

**Results:**

The PD-L1 expression on corresponding immune cells has no significant difference between these two groups. We showed the downregulated PD-1 expression in type 2 diabetes patients. The correlation analysis indicated that the PD-1 on NK cells has a positive correlation with insulin and diabetes duration. And an inverse correlation has been shown between the PD-1 expression on monocytes and BMI (body mass index).

**Conclusions:**

The results in this article suggest that PD-1, unlike PD-L1, might participate in the progression of type 2 diabetes. This investigation will provide evidence for the potential immune therapy for T2D.

## 1. Introduction

Type 2 diabetes (T2D), which affects around 400 million people with high morbidity and mortality, has become a global epidemic problem [1]. Recent investigations showed that T2D is recognized as a chronic, low-grade inflammatory disease in which the expression or activation of the immune-related molecules is altered [2, 3]. Some significant immune molecules, like TLR-3 [4], cytokine interleukin-1*β* [5], and Tim-3 [6], play an important role in the pathogenesis of T2D and its complications. In addition, immune cells, like CD8^+^ and CD4^+^ T cells [7], NK cells [8], and macrophages [9], also take part in T2D-related inflammation.

Programmed death-1 (PD-1), which is a negative regulatory element, is a transmembrane protein that is frequently expressed on T lymphocytes [10]. PD-L1, PD-1's ligand, as stated in the name, is expressed on macrophages and DCs and is regulated upon their activation [11]. PD-1 binding to PD-L1 forms an immunological checkpoint, which can induce and maintain the tolerance of the peripheral T cell [12]. The PD-1/PD-L1 pathway is critical to immune homeostasis and has been reported to be involved in many diseases [12]. And the anticancer therapy which aims at the PD-1/PD-L1 pathway has been illuminated [13]. However, few studies have focused on the PD-1 and PD-L1 expressions in T2D patients.

Here, in this article, we focus on the expressions of the PD-1 and PD-L1 on partial immune cells in T2D patients. The expression of the PD-L1 on CD4^+^ T cells, CD8^+^ T cells, NK cells, and monocytes has no significant difference between the healthy donors and the T2D patients, whereas T2D patients have a much lower expression of PD-1 compared with the healthy donors. This investigation will provide evidence for the potential immune therapy for T2D.

## 2. Methods

### 2.1. Human Blood Samples

23 T2D cases from the Endocrinology Department (Qilu Hospital, Jinan, Shandong, China) were selected for obtaining the peripheral blood. Fasting blood glucose (FPG) ≥ 7.0 mmol/L and/or random blood glucose (RBG) ≥ 11.1 mmol/L are the diagnostic basis for T2D. 20 healthy donors from Shandong Cancer Hospital and Institute were selected for obtaining the peripheral blood. The individuals were negative for hepatitis B virus or other virus infections and specific cases before sampling. This study had been approved by the medical ethics committee of Shandong University, and informed consent was implemented before it was initiated. All the data of the T2D patients and healthy donors are summarized in Table 1.

### 2.2. Flow Cytometry

The peripheral blood mononuclear cells were isolated by centrifuging the whole blood with an EZ-Sep TM (Dakewe, Shenzhen, China) lymphocyte separation tube. The cells were stained with corresponding anti-human FCM antibodies which include anti-PD-1 (eBioscience), anti-PD-L1 (eBioscience), anti-CD8 (eBioscience), anti-CD4 (eBioscience), anti-CD3 (BioLegend), anti-CD14 (eBioscience), and anti-human CD56 (eBioscience). One test contains 10,000 cells which were incubated for 30 min with corresponding antibodies and analyzed by FACSAria II. The gated strategy is shown in Figure S1.

### 2.3. Statistical Analysis

For the data analysis, the Prism GraphPad software (version 6.0) was used. The comparison between the T2D patient group and the healthy donor group was calculated by unpaired two-tailed Student *t*-test, and the correlation coefficient was calculated by Pearson's correlation. *P* < 0.05 was considered a significant difference.

## 3. Results

### 3.1. The Expression of PD-L1 and PD-1 on CD4^+^ T Cells

**Figure 1:**
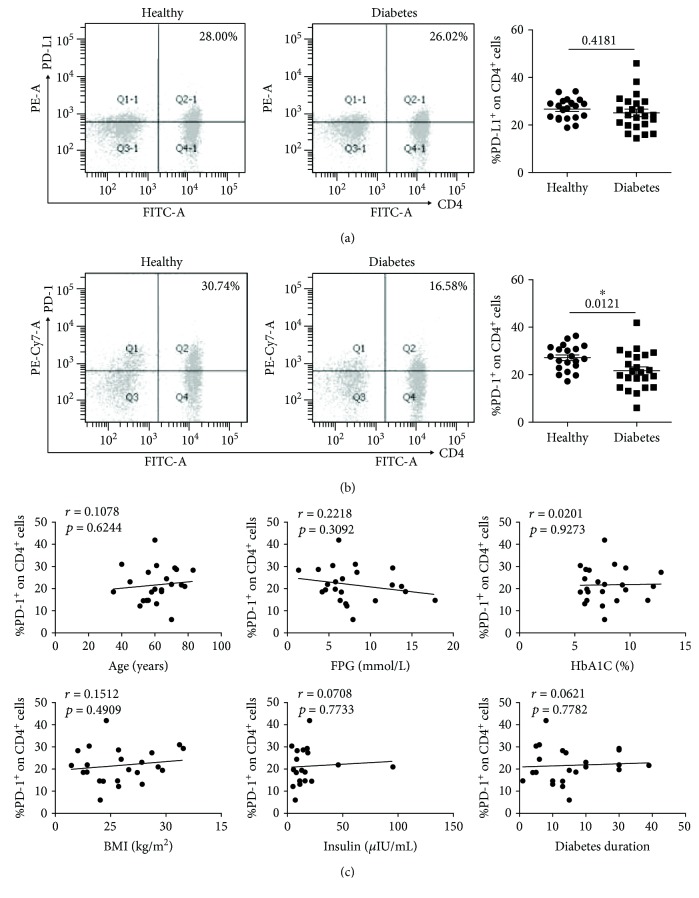
The expressions of PD-L1 and PD-1 on CD4^+^ T cells (marked with CD3^+^CD4^+^) in T2D patients and healthy donors. Peripheral blood mononuclear cells (PBMCs) were isolated from healthy donors (*n* = 20) and patients with T2D (*n* = 23). (a) Typical flow cytometry analysis of PD-L1 expression on CD4^+^ T cells (left) and the statistical graph (right) are shown for the T2D patients (*n* = 23, 25.11 ± 1.57%) and healthy donors (*n* = 20, 26.67 ± 0.98%). (b) Typical flow cytometry analysis of the PD-1 expression on CD4^+^ T cells (left) and the statistical graph (right) are shown for the T2D patients (*n* = 23, 25.11 ± 1.57%) and healthy donors (*n* = 20, 26.67 ± 0.98%). (c) Correlation analysis of the PD-1 expression on CD4^+^ T cells and age, fasting plasma glucose (FPG), glycated hemoglobin (HbA1C), body mass index (BMI), insulin, and diabetes duration. ^∗^ *P* < 0.05.

We first analyzed the expressions of PD-L1 and PD-1 on CD4^+^ T cells (marked with CD3^+^CD4^+^) from the T2D patients and the healthy donors. As shown in Figure 1(a), the PD-L1 expression has no significant difference between the T2D patients (*n* = 23, 25.11 ± 1.57%) and the healthy donors (*n* = 20, 26.67 ± 0.98%). However, the CD4^+^ T cells from the T2D patients (*n* = 23, 21.69 ± 1.64%) display much less PD-1 compared with those from the healthy donors (*n* = 20, 27.15 ± 1.19%) (Figure 1(b), *P* = 0.0121). Since age, body mass index (BMI), diabetes duration, fasting plasma glucose (FPG), glycated hemoglobin (HbA1C), and insulin are important indicators in the progression of T2D, the correlation between the PD-1 expression and these indicators was evaluated. As shown in Figure 1(c), there was no significant correlation between the PD-1 expression and these indicators in CD4^+^ T cells.

### 3.2. The Expression of PD-L1 and PD-1 on CD8^+^ T Cells

**Figure 2 fig2:**
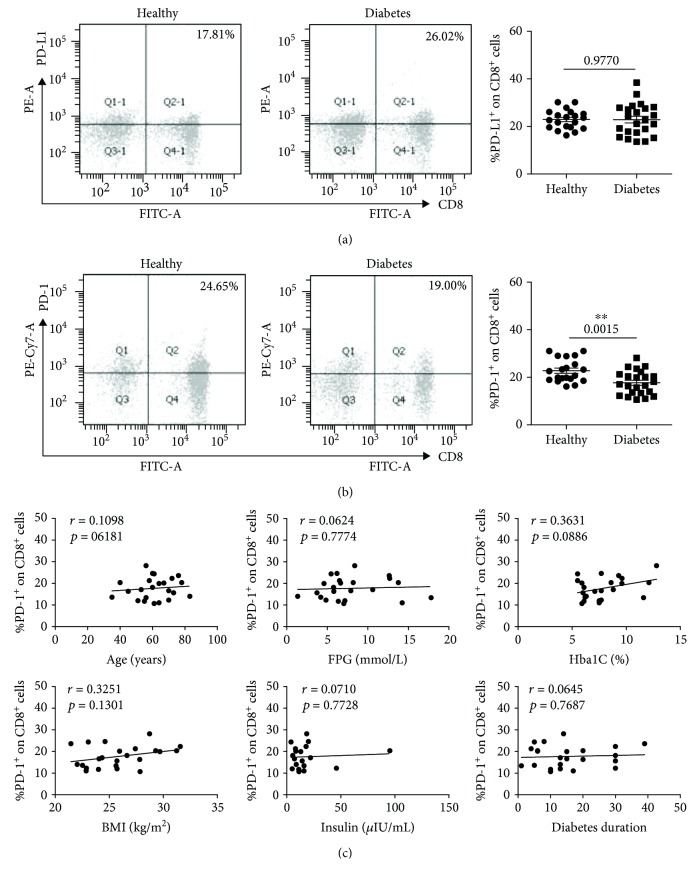
The expressions of PD-L1 and PD-1 on CD8^+^ T cells (marked with CD3^+^CD8^+^) in T2D patients and healthy donors. (a) Typical flow cytometry analysis of the PD-L1 expression on CD8^+^ T cells (left) and the statistical graph (right) are shown for the T2D patients (*n* = 23, 22.88 ± 1.39%) and healthy donors (*n* = 20, 22.93 ± 0.90%). (b) Typical flow cytometry analysis of the PD-1 expression on CD8^+^ T cells (left) and the statistical graph (right) are shown for the T2D patients (*n* = 23, 17.73 ± 1.04%) and healthy donors (*n* = 20, 22.82 ± 1.08%). (c) Correlation analysis of the PD-1 expression on CD8^+^ T cells and age, fasting plasma glucose (FPG), glycated hemoglobin (HbA1C), body mass index (BMI), insulin, and diabetes duration. ^∗∗^ *P* < 0.01.

At the same time, the expressions of PD-L1 and PD-1 on CD8^+^ T cells (marked with CD3^+^CD8^+^) were analyzed. The CD8^+^ T cells display the same PD-L1 and PD-1 expression tendency with CD4^+^ T cells. There was no significant PD-L1 expression difference between the T2D patients (*n* = 23, 22.88 ± 1.39%) and the healthy donors (*n* = 20, 22.93 ± 0.90%) (Figure 2(a)). The PD-1 expression was downregulated in the T2D patients (*n* = 23, 17.73 ± 1.04%) compared with the healthy donors (*n* = 20, 22.82 ± 1.08%) (Figure 2(b), *P* = 0.0015). Just like the CD4^+^ T cells, the correlation analysis showed no significance in CD8^+^ T cells (Figure 2(c)).

### 3.3. The Expression of PD-L1 and PD-1 on NK Cells

**Figure 3 fig3:**
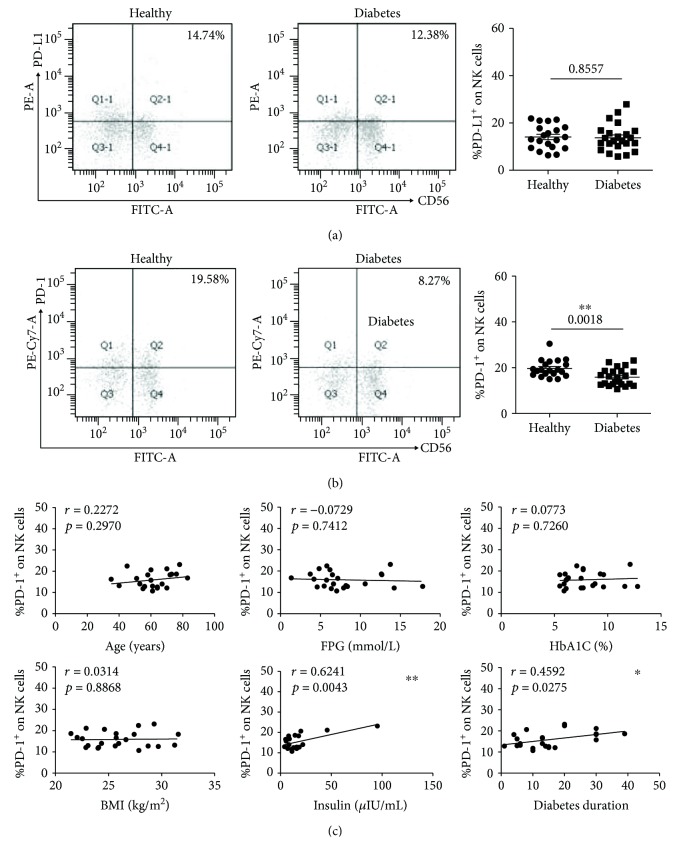
The expressions of PD-L1 and PD-1 on NK cells (marked with CD3^−^CD56^+^) in T2D patients and healthy donors. (a) Typical flow cytometry analysis of the PD-L1 expression on NK cells (left) and the statistical graph (right) are shown for the T2D patients (*n* = 23, 13.37 ± 1.19%) and healthy donors (*n* = 20, 14.04 ± 1.13%). (b) Typical flow cytometry analysis of the PD-1 expression on NK cells (left) and the statistical graph (right) are shown for the T2D patients (*n* = 23, 15.89 ± 0.77%) and healthy donors (*n* = 20, 19.68 ± 0.83%). (c) Correlation analysis of the PD-1 expression on NK cells and age, fasting plasma glucose (FPG), glycated hemoglobin (HbA1C), body mass index (BMI), insulin, and diabetes duration. ^∗^ *P* < 0.05 and ^∗∗^ *P* < 0.01.

NK cells were marked with CD3^−^CD56^+^ in the FCM detection. The healthy donors (*n* = 20, 14.04 ± 1.13%) have almost the same PD-L1 expression on NK cells as the T2D patients (*n* = 23, 13.37 ± 1.19%) (Figure 3(a)). However, as shown in Figure 3(b), the T2D patients (*n* = 23, 15.89 ± 0.77%) have a much lower level of PD-1 expression compared with the healthy donors (*n* = 20, 19.68 ± 0.83%). A positive correlation was found between the PD-1 expression and insulin (Figure 3(c), *r* = 0.6241, *P* = 0.0043) and diabetes duration (Figure 3(c), *r* = 0.4592, *P* = 0.0275) in the correlation analysis. The other indicators had no correlation with the expression of PD-1 on NK cells.

### 3.4. The Expression of PD-L1 and PD-1 on Monocytes

**Figure 4:**
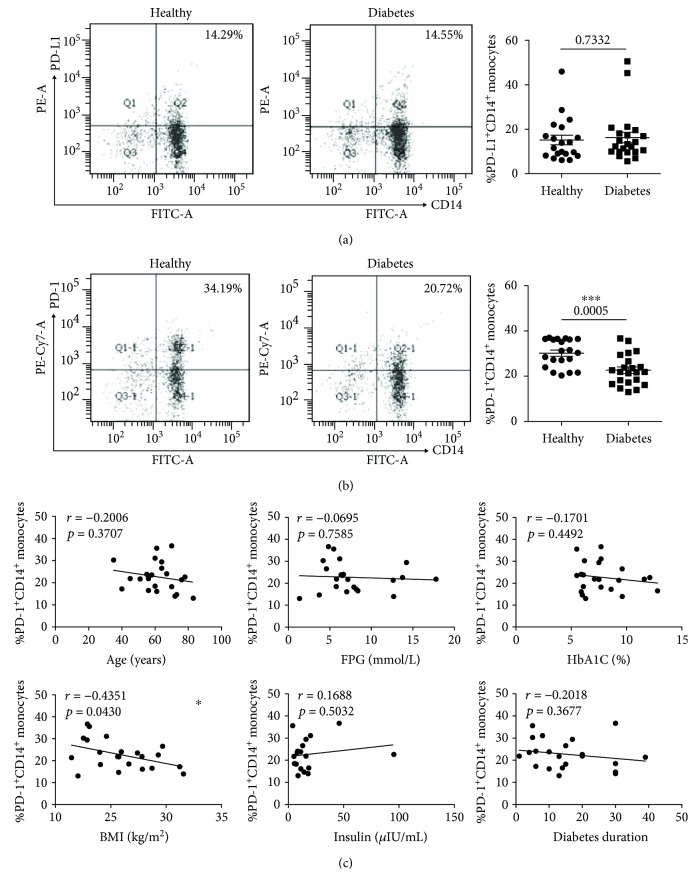
The expressions of PD-L1 and PD-1 on monocytes (marked with CD14^+^) in T2D patients and healthy donors. (a) Typical flow cytometry analysis of the PD-L1 expression on monocytes (left) and the statistical graph (right) are shown for the T2D patients (*n* = 22, 16.28 ± 2.38%) and healthy donors (*n* = 20, 15.17 ± 2.16%). (b) Typical flow cytometry analysis of the PD-1 expression on monocytes (left) and the statistical graph (right) are shown for the T2D patients (*n* = 22, 22.67 ± 1.43%) and healthy donors (*n* = 20, 30.16 ± 1.35%). (c) Correlation analysis of the PD-1 expression on monocytes and age, fasting plasma glucose (FPG), glycated hemoglobin (HbA1C), body mass index (BMI), insulin, and diabetes duration. ^∗^ *P* < 0.05 and ^∗∗∗^ *P* < 0.001.

At the end, we analyzed the expressions of PD-L1 and PD-1 on monocytes (marked with CD14^+^). The expression of PD-L1 on monocytes still has no significant difference between these two groups (healthy donors, *n* = 20, 15.17 ± 2.16%; T2D patients, *n* = 22, 16.28 ± 2.38%) (Figure 4(a)). The PD-1 expression on monocytes from T2D patients (*n* = 22, 22.67 ± 1.43%) was significantly lower than that from healthy donors (*n* = 20, 30.16 ± 1.35%) (Figure 4(b)). In the correlation analysis, except for the BMI (*r* = −0.4351, *P* = 0.0430) which has an inverse correlation with the PD-1 expression, the other indicator had no correlation with the expression of PD-1 (Figure 4(c)).

## 4. Discussion

Programmed death-1 (PD-1), which is a member of the B7-CD28 family, has been identified as a negative costimulatory immune molecule [14, 15]. PD-1 can be expressed on some immune cells, like T cells, NKs, B cells, and monocytes. PD-L1 which is the ligand for PD-1 is expressed on DCs, macrophages, vascular endothelial cells, and so on [11]. The PD-1/PD-L1 signaling pathway balances the stimulatory and inhibitory signals needed for effective immune responses to microbes and maintenance of self-tolerance, respectively [12]. Studies have confirmed that the PD-1/PD-L1 signaling pathway is connected with many diseases closely, such as cancer, particularly non-small-cell lung cancer [16], and autoimmune diseases, SLE (systemic lupus erythematosus) [17], T1D [18], and rheumatoid arthritis [19]. So the PD-1/PD-L1 has been proved to be the important target in the treatment of corresponding diseases.

With regard to T2D which is characterized by chronic, low-grade inflammation, amounts of evidence show that the related immune cells take part in the pathogenetic procession of T2D. In this article, we detected the PD-1 and PD-L1 expressions on these immune cells in T2D patients aiming to investigate their potential therapeutic action in the T2D progression. The results indicated that the PD-L1 expression has no obvious difference between the T2D patients and healthy donors (Figures 1 [Fig fig2] [Fig fig3]–4(a)). However, unlike PD-L1, PD-1 is reduced on these cells in T2D patients. Accordingly, serum of T2D patients includes soluble costimulators and cytokines, which might affect the function and expression of PD-1.

There is a report indicating that the dysregulation of CD4^+^CXCR5^+^ T cells plays vital roles in the progression of T2D [20]. And the CD4^+^ T cells which come from the peripheral blood frequently consistently correlates with an increased body mass index or adiposity [21]. There is a report which showed that the T2D patients have an increased expression of PD-1 on CD4^+^CD28^−^ cells [22]. But there is also evidence which shows that the T2D patients and the healthy donors have the same level of PD-1 on CD4^+^ T cells [23]. All the above indicate that the PD-1 on CD4^+^ T cells may function in T2D. We analyzed the PD-1 expression on CD4^+^ T cells and found that PD-1 was highly induced on CD4^+^ T cells from healthy controls but not from T2D patients (Figure 1(b)). The low expression of PD-1 may generate aberrant T cells which can function in T2D progression.

The reactivity of CD8^+^ T cells to islet antigens is unique and has been reported in T1D [24], while Nishimura et al. showed that CD8^+^ T cells are responsible for macrophage activation and recruitment into adipose tissue of obese mice in the progression of T2D [25]. The PD-1 expressed on CD8^+^ T cells can mediate the exhaustion of CD8^+^ T cells. In the current report, we found the downregulation of PD-1 on CD8^+^ T cells in T2D patients (Figure 2(b)) which indicates that macrophages were recruited much more into adipose tissue that was activated by CD8^+^ T cells.

The count of NK cells has been reported to be increased in T2D patients [26]. And the function of natural killer cells, which as an important target for infection and tumor protection, is impaired in T2D [27]. The upregulation of PD-1 on NK cells can mediate the exhaustion of activated NK cells in tumor immunity [28]. The expression of PD-1 in T2D patients has not been illustrated. We detected the PD-1 on NK cells in T2D patients and found it had a much lower level than in healthy donors (Figure 3(b)). The decreased PD-1 expression may be attributed to the increased NK cell numbers.

Compared with the immune cells above, monocytes which are related to adipose tissue inflammation play an important role in T2D. The monocytes infiltrate into the adipose tissue and turn into adipose tissue macrophages which have been distinguished as the proinflammatory phenotype M1 and anti-inflammatory phenotype M2. M2 carry out the adipose tissue remodeling functions and surveillance, and M2 are important in the insulin sensitivity maintenance [29]. A previous study has shown that PD-1 induces the macrophage's M2 polarization [30]. The present study showed that the monocytes which were isolated from T2D patients have a lower expression of PD-1 (Figure 4(b)) which indicate that T2D patients display chaotic insulin sensitivity.

In conclusion, the expression of the PD-L1 on CD4^+^, CD8^+^, NK cells, and monocytes has no significant difference between the T2D patients and the healthy donors, whereas PD-1 is downregulated in the T2D patients. Our data suggest that PD-1 that expressed on these immune cells might participate in the progression of T2D. However, the specific mechanism of the PD-1 expression regulation and the potential role of PD-1 in the progression of T2D still need further investigation.

## Figures and Tables

**Table 1:** Clinical characteristics of T2D patients and healthy donors.

Characteristics	T2D patients (*n* = 23)	Healthy donors (*n* = 20)
Female, *n* (%)	13 (56.52)	10 (50.00)
Age (years) (range)	61 (35–78)	57 (42–68)
BMI (kg/m^2^)	25.87 ± 0.76	21.56 ± 0.57
HbA1c (%)	9.02 ± 0.57	5.93 ± 0.94
FPG (mmol/L)	8.91 ± 1.02	4.42 ± 0.78
Insulin (*μ*IU/mL)	20.32 ± 7.95	11.54 ± 6.32
Diabetes duration (years)	15.39 ± 4.48	—

Data are expressed as median ± standard error of the mean. BMI: body mass index; FPG: fasting plasma glucose; HbA1c: glycated hemoglobin; T2D: type 2 diabetes.

## Data Availability

No data were used to support this study.

## Supplementary Materials

Supplementary Materials Figure S1: gated strategy of FCM analysis: (a) gated strategy of CD4^+^ T cells, CD8^+^ T cells, and NK cells (Figures 1 [Fig fig2]–3); (b) gated strategy of CD14^+^ monocytes (Figure 4).
