# Supplementary material for: Unlike PD-L1, PD-1 Is Downregulated on Partial Immune Cells in Type 2 Diabetes
Source: J Diabetes Res. 2019 Mar 17;2019:5035261. doi: 10.1155/2019/5035261 (PMC6441514; doi:10.1155/2019/5035261)
Supplement: Supplementary Materials — Figure S1: gated strategy of FCM analysis: (a) gated strategy of CD4+ T cells, CD8+ T cells, and NK cells (Figures 1 –3); (b) gated strategy of CD14+ monocytes (Figure 4). [file 5035261.f1.doc]

**Figure S1**

**
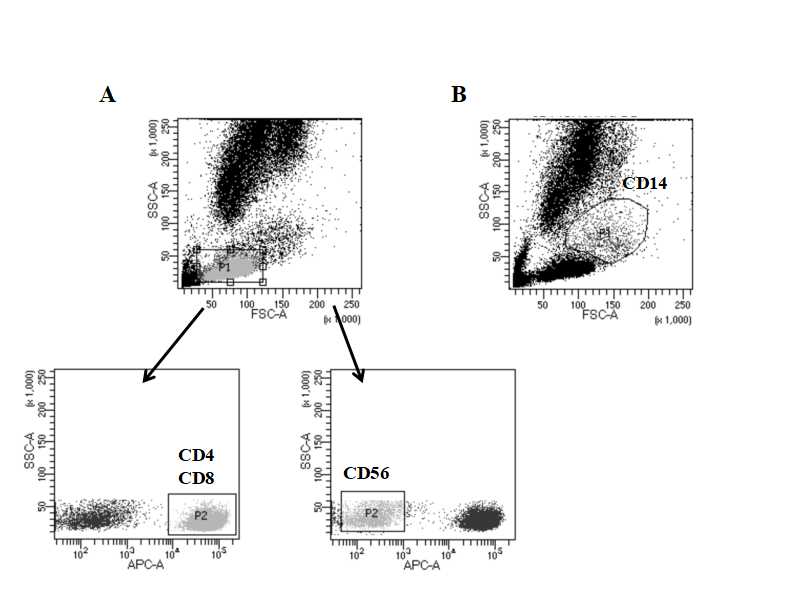
**

**Figure S1. Gated strategy of FCM analysis.** (A) Gated strategy of CD4^+^ T cells, CD8^+^ T cells, NK cells (Figure 1, 2 and 3). (B) Gated strategy of CD14^+^ monocytes (Figure 4).
